# Propyl-phthalimide Cyclotricatechylene-Based Chemosensor for Sulfosulfuron Detection: Hybrid Computational and Experimental Approach

**DOI:** 10.1021/acsomega.3c05510

**Published:** 2023-10-24

**Authors:** Nihal Patel, Krunal Modi, Keyur Bhatt, Jaymin Parikh, Ajay Desai, Bhavesh Jain, Nirali Parmar, Chirag N. Patel, Alan Liska, Jiri Ludvik, Shibu Pillai, Brij Mohan

**Affiliations:** † Department of Chemistry, Faculty of Science, Ganpat University, Kherva, Mehsana, Gujarat 384012, India; ‡ Department of Humanity and Sciences, Indrashil University, Kadi, Mehsana, Gujarat 382740, India; § Department of Computer Science and Engineering, Indrashil University, Kadi, Mehsana, Gujarat 382740, India; ∥ Department of Botany, Bioinformatics and Climate Change Impacts Management, School of Science, Gujarat University, Ahmedabad, Gujarat 380009, India; ⊥ Biotechnology Research Center, Technology Innovation Institute, Abu Dhabi 9639, United Arab Emirates; # Department of Molecular Electrochemistry and Catalysis, J. Heyrovsky Institute of Physical Chemistry, Academy of Sciences of the Czech Republic, Dolejskova 2155/3,182 23 Praha 8, Czech Republic; ∇ Department of Chemistry, Institute of Technology, Nirma University, Ahmedabad, Gujarat 380009, India; ○ Centro de Química Estrutural, Institute of Molecular Sciences, Instituto Superior Técnico, Universidade de Lisboa, Av. Rovisco Pais, 1049-001 Lisboa, Portugal

## Abstract

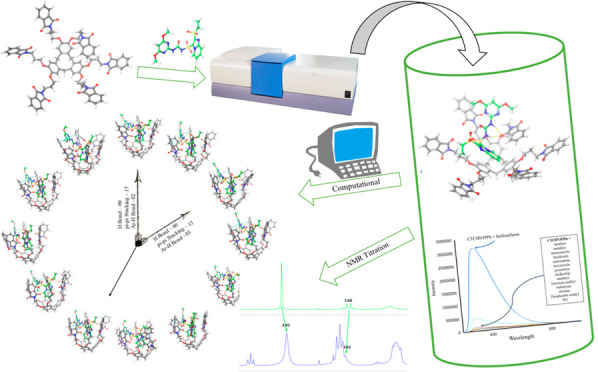

The detection of trace amounts of sulfosulfuron, a pesticide of increasing importance, has become a pressing issue, prompting the development of effective chemosensors. In this study, we functionalized cyclotricatechylene (CTC) with propyl-phthalimide due to the presence of electronegative oxygen and nitrogen binding sites. Our optimized ligand displayed the highest docking score with sulfosulfuron, and experimental studies confirmed a significant fluorescence enhancement upon its interaction with sulfosulfuron. To gain a deeper understanding of the binding mechanism, we introduced density functional theory (DFT) studies. We carried out binding constant, Job’s plot, and limit of detection (LOD) calculations to establish the effectiveness of our chemosensor as a selective detector for sulfosulfuron. These findings demonstrate the potential of our chemosensor for future applications in the field of pesticide detection.

## Introduction

Detecting the presence of pesticides in the environment is crucial to prevent exposure and ensure safety.^1−4^ Pesticides can have a negative impact on the environment, including water pollution, soil degradation, and harm to wildlife. Sensors can help to monitor the levels of pesticides in the environment and provide data for scientists and policymakers to make informed decisions about pesticide use.^5−7^ Pesticides are widely used in agriculture to protect crops from pests and disease. However, the excessive use of pesticides can lead to crop damage and harm to beneficial organisms. Sensors can help farmers monitor pesticide levels and optimize their use, reducing waste and minimizing the environmental impact.^7,8^ There are regulations in place to ensure that pesticides are used safely and responsibly. Sensors can provide data to ensure that these regulations are being followed and that pesticide use is within acceptable limits.^9−11^ Sensors for pesticides are important for safety, environmental monitoring, agricultural optimization, and regulatory compliance. By detecting and monitoring pesticide levels, we can minimize the negative impacts of these substances on human health and the environment.

**Figure 1:**
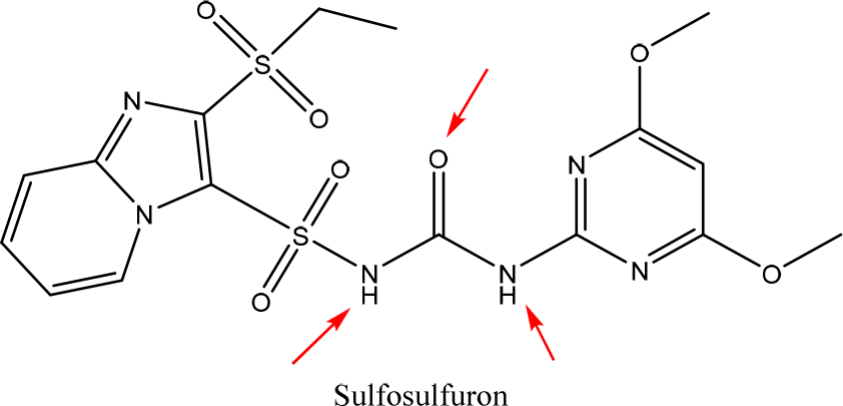
Structure of sulfosulfuron (SS).

Sulfosulfuron (SS) (Figure 1) is an herbicide used in agriculture to control weeds in crops such as wheat, corn, and rice. It is effective and selective but can have negative environmental effects if not used properly. It can be carried by rainwater or irrigation runoff, us toxic to aquatic organisms, depletes soil nutrients, and harms nontarget plant species and beneficial organisms. Chemical sensors detect sulfosulfuron levels in water, soil, and other environmental samples, enabling better monitoring and regulation for safe and responsible herbicide use.^12−14^

Fluorescence sensing is a selective chemical sensor technique for detecting and quantifying target molecules. It measures light emission from excited fluorescent molecules to determine concentration in samples and is particularly useful for small molecules in environmental pollutants or biological fluids. It can also be used to detect changes in the environment, such as changes in pH or temperature, by monitoring changes in the fluorescence signal. Overall, fluorescence sensing is a powerful tool for chemical sensing and has many applications in fields such as environmental monitoring, biomedical diagnostics, and food safety.^15−18^

Cyclotriveratrylene (CTV) is a trimeric molecule studied for potential pesticide sensing due to its hydrophobic cavity and potential optical detection of properties through fluorescence or absorbance spectroscopy.^19^ Supramolecules like CTV offer unique chemical sensing properties, enabling selective binding to target molecules and thus the development of highly sensitive and specific sensors.^20−22^ Supramolecules can also be used to create sensor arrays, which consist of multiple sensors that each respond differently to a range of target molecules. By analyzing the pattern of responses from the array, it is possible to identify and quantify the target molecules in a sample. This approach can be used for the detection of complex mixtures, such as in environmental monitoring or food safety applications.^23,24^ Overall, supramolecules like CTV have great potential for use in chemical sensing due to their unique properties and ability to selectively bind to target molecules. Their use in chemical sensing can lead to the development of sensitive, selective, and reliable sensors for a wide range of applications.^19,25−27^

In this research, the new hexaphthalimide-functionalized cyclotricatechylene derivative (CTCHN3PPh) was synthesized and tested for its ability to detect the presence of the sulfosulfuron pesticide in dimethylformamide (DMF). The ligand was found to exhibit a significant increase in emission intensity in the presence of the sulfosulfuron pesticide compared with other pesticides tested. The electron-withdrawing nature of the phthalimide group enhances the binding of the ligand to the pesticide. Additionally, the three-dimensional structure of the cyclotriveratrylene derivative may allow for specific interactions with the pesticide molecule, resulting in increased fluorescence intensity. Further studies, such as fluorescence titration and computational modeling, can help to better explain the nature of this interaction.

## Results and Discussion

### Design of the Ligand

Sulfonylureas, like sulfosulfuron, comprise both a sulfonyl group and a urea group, whereas propyl-phthalimide consists of a phthalimide group and a propyl group. The phthalimide group is a cyclic imide group that contains two carbonyl groups and a nitrogen atom. While it is feasible for sulfonylureas and propyl-phthalimide to interact with each other, they may react to form a salt or form a complex due to hydrogen bonding between the nitrogen and oxygen atoms of the phthalimide and urea groups, respectively. Furthermore, the solubility and pH of the solution can also influence the reactivity of the compounds. In a related context, propyl-phthalimide-functionalized cyclotricatechylene was synthesized using a conventional method, then the synthesized ligand and analytes were optimized using Gaussian software with the Becke’s three-parameter hybrid exchange (B3LYP) method and the hybrid functional using the Coulomb attenuating method (CAM-B3LYP) with 6-31G(d,p) and 6-311G(d,p) basis sets; the optimization energy is reported in Table 1. This evidence indicates that the reactivity and potential interactions of propyl phthalimide with other molecules can be effectively studied by using computational methods.

**Table 1:** Geometry Optimization and Complexation Energy of CTCHN3PPh

basis set	method	gas	solvent	difference
b3lyp	6-31g(d,p)	–5041.47	–5041.530732	0.056287
b3lyp	6-311g(d,p)	–5042.63	–5042.686911	0.061166
cam b3lyp	6-31g(d,p)	–5039.02	–5039.077282	0.056317
cam b3lyp	6-311g(d,p)	–5040.19	–5040.252315	0.060544

### General Synthetic Procedure for CTCHN3PPh (2,2′,2″,2‴,2⁗,2⁗′-(((10,15-Dihydro-5*H*-tribenzo[*a*,*d*,*g*][9]annulene-2,3,7,8,12,13-hexayl)hexakis(oxy))hexakis(propane-3,1-diyl))hexakis(isoindoline-1,3-dione)) (CTCHN3PPh)

Cyclotricatechylene (CTC) was synthesized using the method first reported with the demethylation of cyclotriveratrylene^28,29^ (Scheme S1, Figures S1–S3). To a stirred solution of cyclotricatechylene (CTC) (0.5 g, 1.36 mmol, 1.0 equiv) in DMF (10 mL) were added *N*-(3-propyl)phthalimide (N3PPh) (2.56 g, 9.56 mmol, 7.0 equiv), K_2_CO_3_ (1.31 g, 9.56 mmol, 7.0 equiv), and KI (0.022 g, 0.13 mmol, 0.1 equiv). The reaction mixture was heated at 70 °C for 3–4 h. After the completion of the reaction, it was quenched with water, and the product was extracted in DCM (50 mL × 3). The combined organic layer was dried under vacuum, and the product was purified by flash chromatography using 10–15% ethyl acetate/hexane as the eluent, resulting in the isolation of a pure compound (0.12 g, 0.080 mmol). *m*/*z* (ESI-MS) 1487.75 [M – 1]^+^ (Figure S5); NMR 24H (Ar–H) 7.83–7.84 m, 12H (CH_2_) 3.46–3.49 (t), 12H (CH_2_) 3.64–3.75 (t), 12H (CH_2_) 1.75–1.81 (p), 6H (Ar–H) 7.52 (s), 3H (CH_2_) 2.75 (s), 3H (CH_2_) 2.90 (s) (Figure S4); FTIR 1699 cm^–1^ (C=O), 1227 cm^–1^ (C–O), 1399 cm^–1^ (C–N) (Figure S6).

**Scheme 1:**
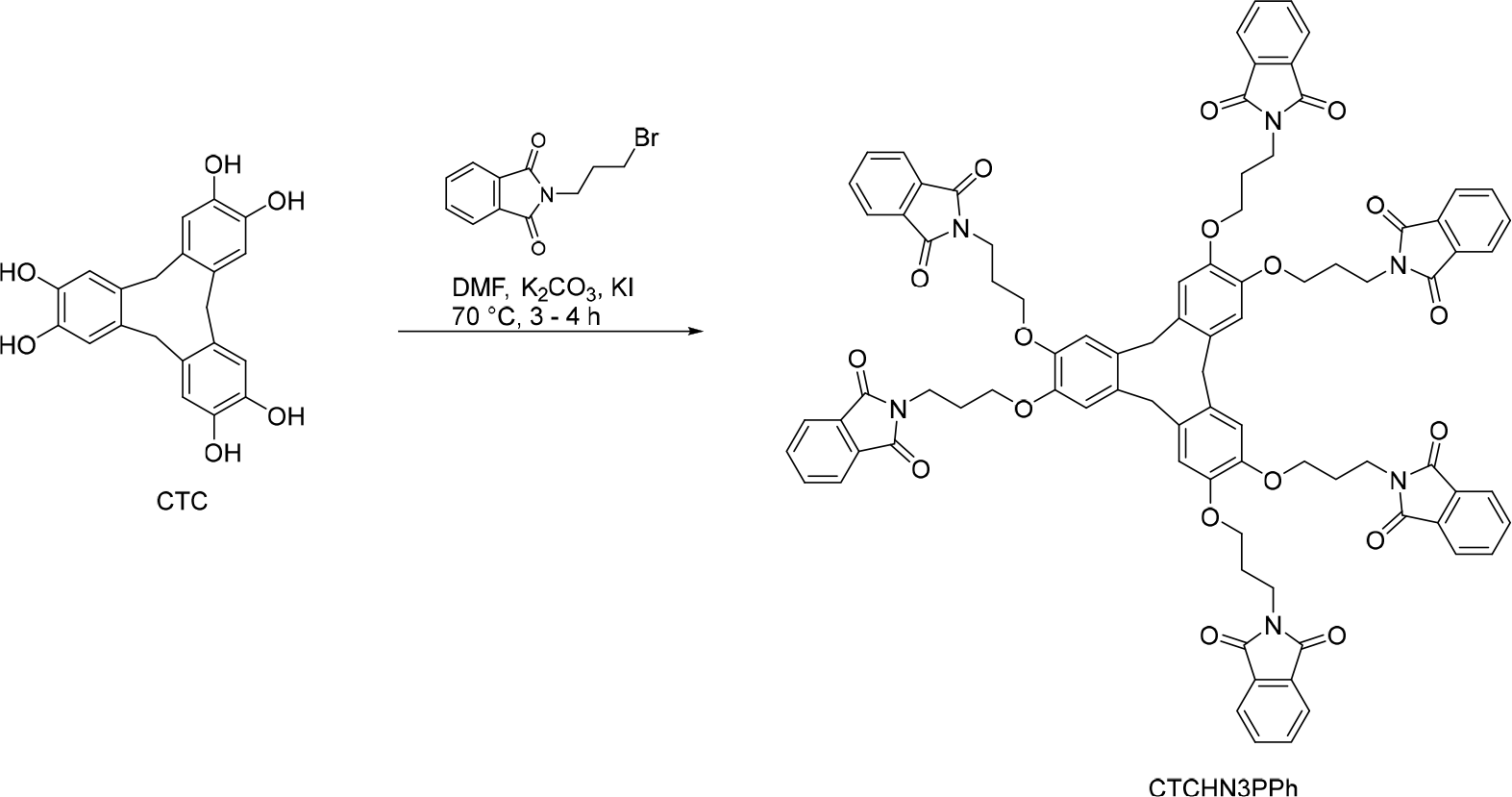
Synthesis of Cyclotricatechylene Hexa-*N*-(3-propyl)phthalimide (CTCHN3PPh)

### Spectrofluorimetry Analysis

**Figure 2:**
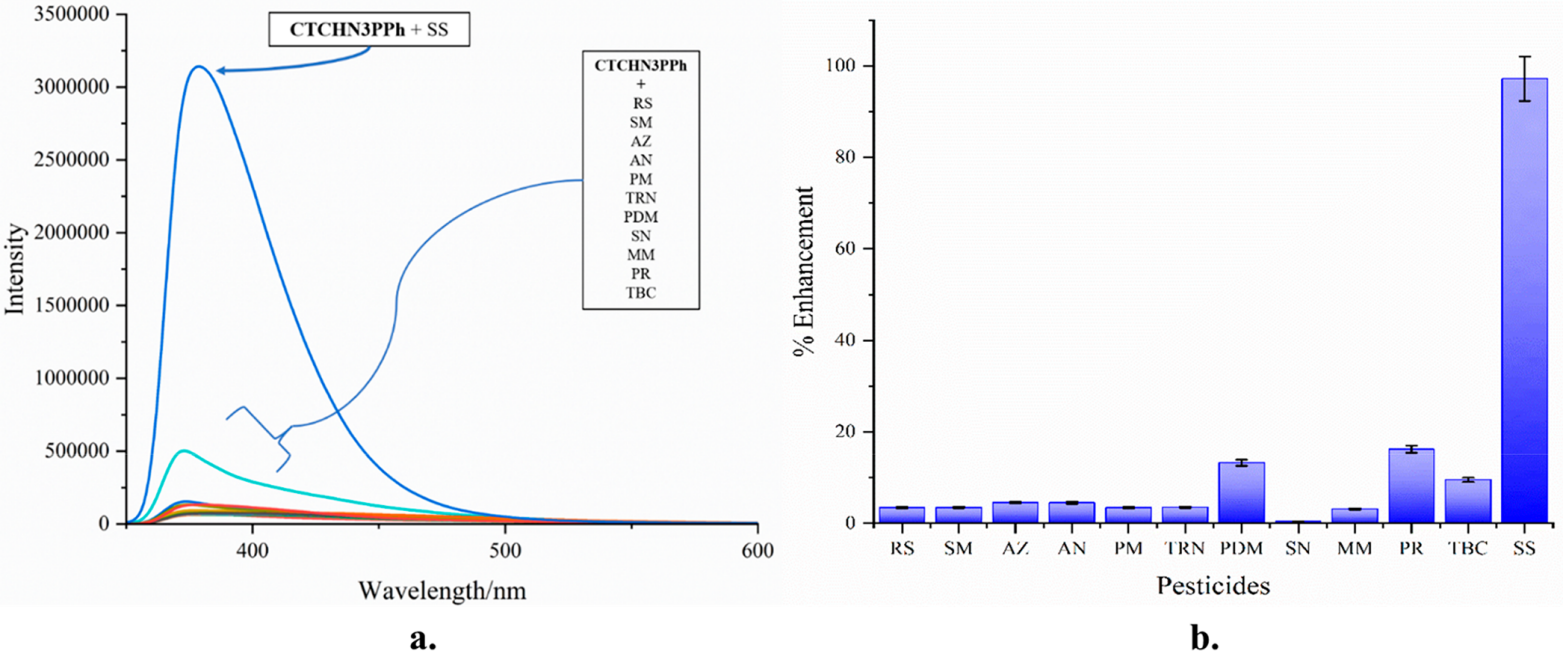
(a) Relative fluorescence enhancement of CTCHN3PPh toward various pesticides. (b) Enhancement percentage (with error) of the fluorescence intensities of the complex thin film for different pesticides.

**Figure 3:**
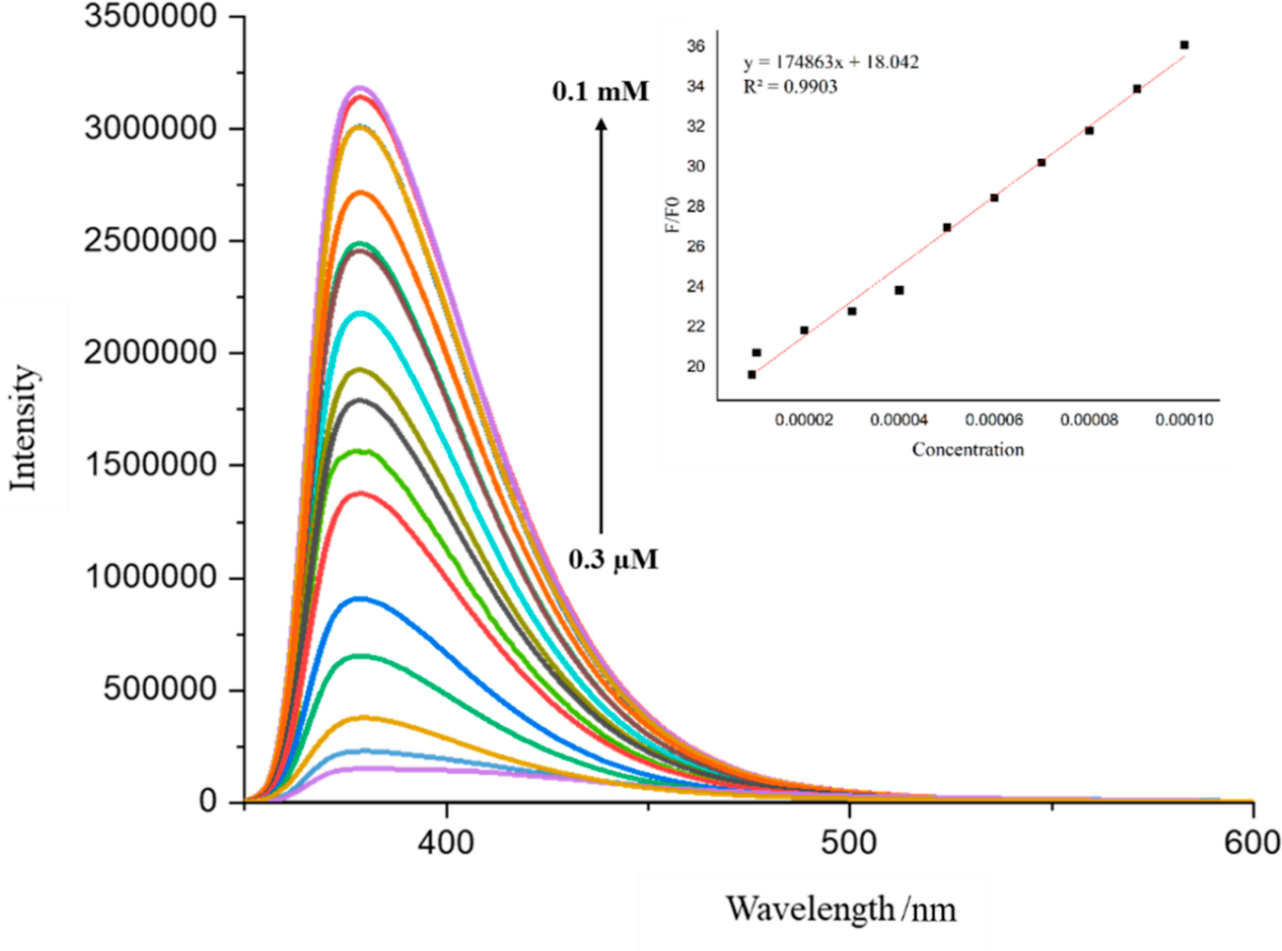
Fluorescence titration of CTCHN3PPh upon the addition of sulfosulfuron solutions. The inset shows the linear regression fit of the titration data as a function of the concentration of SS.

**Figure 4:**
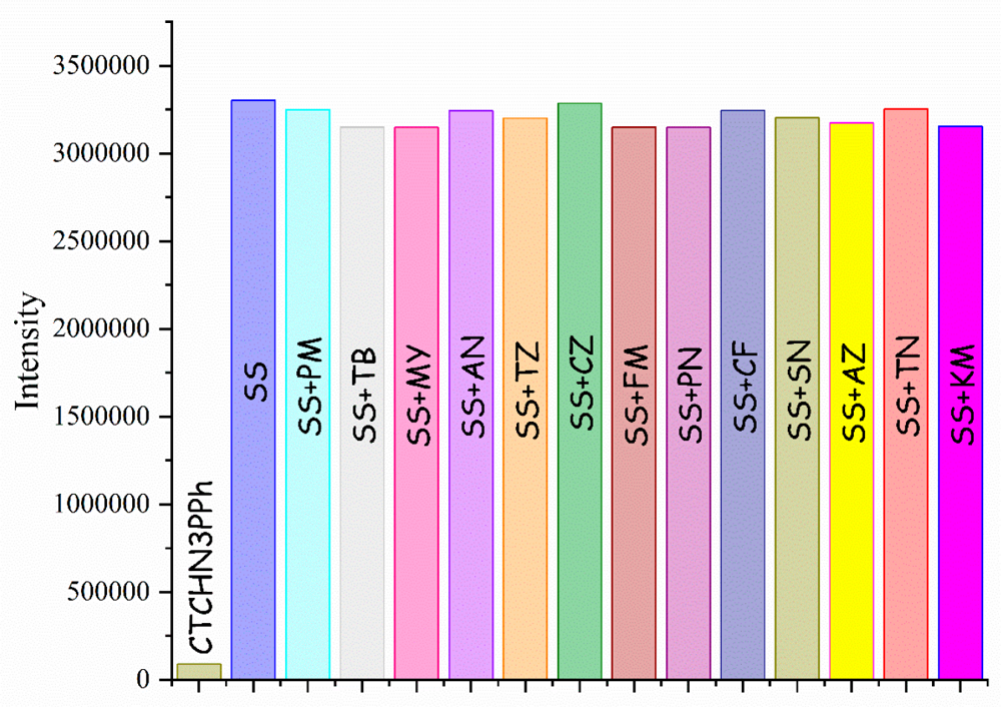
Interference study of the CTCHN3PPh_SS complex with other tested pesticides.

The selectivity of the synthesized CTCHN3PPh probe toward pesticides was evaluated through absorption and emission spectra. The receptor showed an absorption maximum at 293 nm, and no change in the wavelength was observed upon interaction with other pesticides. Emission studies were performed, and fluorescence emission of CTCHN3PPh was observed at 379 nm. Remarkable enhancement of the emission intensity (up to 97% Figure 2(b)) was observed upon the addition of sulfosulfuron (Figure 2(a)), while no significant intensity change was observed during interactions with other pesticides (Figure 2(a)). The fluorescence enhancement may be due to the charge transfer between the HOMO of CTCHN3PPh and the LUMO of sulfosulfuron and the π–π interaction between CTCHN3PPh and sulfosulfuron. Emission titration experiments were carried out with different concentrations of analyte (0.3 μM to 0.1 mM) to gain more insight into the selectivity of the ligand. Job’s plot titrations were also carried out to confirm the stoichiometry of binding. Upon successive addition of sulfosulfuron (SS) to the solution of CTCHN3PPh, a gradual decrease in fluorescent maxima was observed at 379 nm (Figure 3). It was observed that upon decreasing the concentration of the analyte, the fluorescence enhancement gradually decreased (Figure 3). To further affirm the probe’s selectivity, we conducted an interference study of CTCHN3PPh and sulfosulfuron (SS) with the other tested pesticides, such as rimsulfuron (RS), sulfometuron methyl (SM), atrazine (AZ), ametryn (AN), prometryn (PM), terbutryn (TRN), pendimethaline (PDM), simetryn (SN), metsulfuron methyl (MM), propanil (PR), and tebuconazole (TBC). Our findings revealed that there were no substantial alterations in the emission intensity of the complex spectra (Figure 4). This outcome underscores the capability of CTCHN3PPh to reliably detect sulfosulfuron (SS) even in the presence of various analytes, reinforcing its outstanding selectivity for SS within intricate pesticide mixtures.

### Study of Complex

In order to evaluate the binding constant, limit of detection (LOD), and limit of quantification (LOQ) of a pesticide, an emission titration was conducted using a literature procedure.^30,31^ The pesticide in question showed a substantial change in the emissions spectra. The equation used to calculate the binding constant (*K*_s_) is
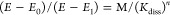
where *E*_0_ and *E*_1_ are the relative fluorescence intensities of the complex without the addition of analyte pesticide and with the maximum concentration of pesticide, respectively, and *n* is the average number of binding sites occupied by a guest molecule. By plotting the graph of log[(*E*_0_ – *E*)/(*E* – *E*_1_)] versus log[*M*], the value of *K*_diss_ is obtained, which is the reciprocal of the binding constant. The titration data yielded a binding constant of 1.7 × 10^5^ M^–1^ with a linear fit (*R*^2^ = 0.9903).

**Figure 5:**
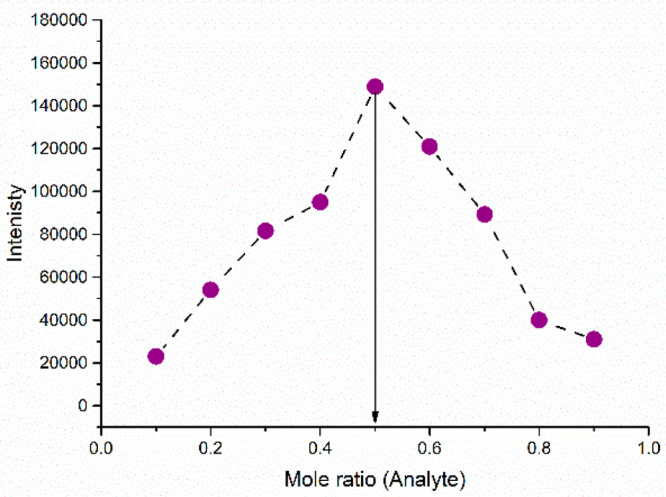
Job’s plot showing the 1:1 stoichiometry for CTCHN3PPh with sulfosulfuron.

The Job’s plot is a graphical technique employed in analytical chemistry to determine the stoichiometry of a complex formed between an analyte and a ligand. This method involves generating a series of solutions with varying concentrations of CTCHN3PPh and sulfosulfuron while keeping the total concentration constant. The measured emission intensity from the blank solution is subtracted from the observed emission intensities of the different concentrations. A value of 0.50 in the resulting plot signifies a 1:1 ratio of the analyte and the ligand (as demonstrated in Figure 5).

### Limit of Detection and Response Time

Limit of detection (LOD) is a measure of the lowest concentration of a substance that can be reliably detected in an analytical measurement.^31,32^ It is an important performance characteristic of analytical methods, as it provides information about the sensitivity of the method and the ability to detect trace levels of a substance. The LOD was calculated using the slope (*m*) and standard deviation (*s*) from a linear calibration curve of different concentrations of sulfosulfuron with respect to the signal intensity (Figure 3). The sensor exhibits a LOD of 58 μM and a limit of quantification (LOQ) of 170 μM, demonstrating its ability to accurately detect and quantify substances within a specified range. Table 2 provides a comprehensive comparison of recently reported calix-based receptors and their corresponding responses toward various analytes, specifically focusing on their interactions with pesticides with phthalimide-functionalized calixarenes.

**Table 2:** Comprehensive Comparison of Recently Reported Calix-Based Receptors

receptor	analyte	technique	binding mechanism	LOD	ref
benzenesulfonyl-tetrasubstituted thiacalix[4]arene	sulfosulfuron (SS)	fluorescence	PET	0.21 ppm	(14)
tetramethoxy resorcinarene tetraquinoline acetamide	*N*-methyl- 4-nitroaniline	UV–vis spectroscopy/fluorescence	π–π interaction		(34)
*meso*-tetraacetohydrazude-calix[4]pyrrole-AuNPs	dimoxystrobin	fluorescence	replacement of the hydrazine group with a pesticide	5 ppm	(35)
bidansylated oxacalix[4]arene	pendimethalin	fluorescence	π-lone pair		(36)
5,17-di(*N*-(9,10-dioxo-9,10-dihydro-anthracen-1-yl)acetamide)tetranitro-oxacalix[4]arene	*N*-methyl-*p*-nitroaniline	fluorescence	charge transfer	0.5 mM	(37)
naphthol-appended calix[4]arene	metolcarb	fluorescence spectroscopy, UV–vis spectroscopy, and FT-IR spectroscopy	structural changes of the calixarene group shell surrounding the NP core upon intercalation	0.08 μM	(38)
thiacalixphenyl[4]arene tetra-*N*-(3-propyl)phthalimide	Hg^2+^	fluorescence	PET	3.10 nM	(39)
oxacalix[4]arene-appended *N*-(3-bromopropyl)phthalimide	4-nitro toluene (4-NT)	fluorescence	PET	2.4 μM	(40)

**Figure 6:**
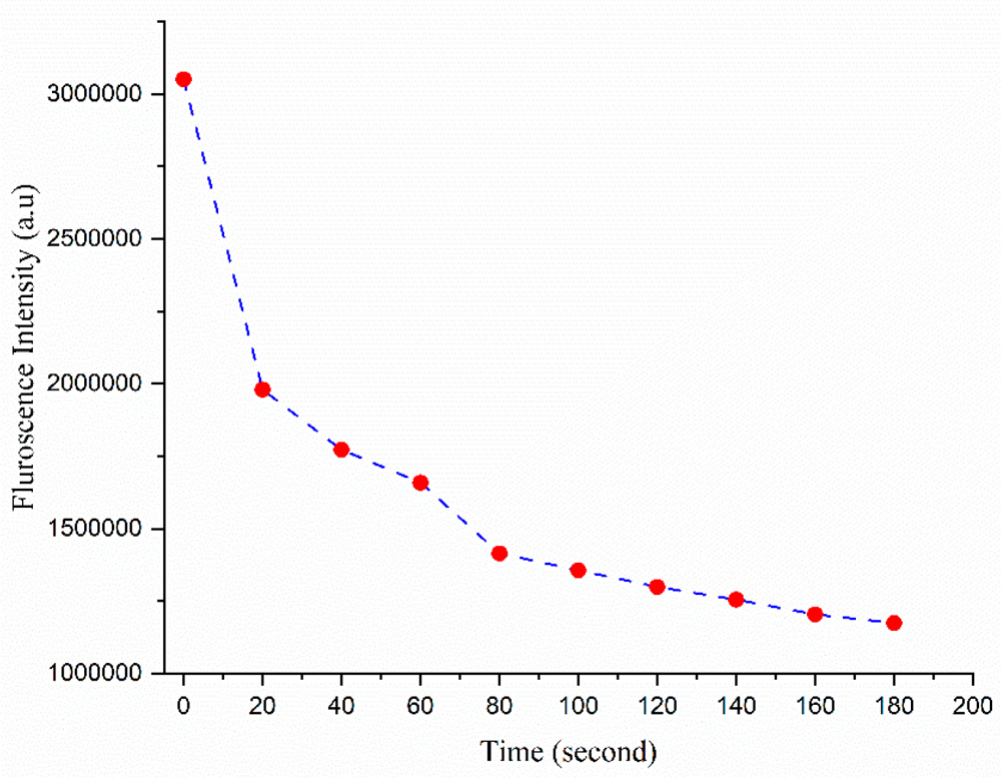
Time dependence study of CTCHN3PPh.

Apart from selectivity and sensitivity, the rapid response of a chemosensor holds significant importance as a parameter.^41^ To assess the sensor’s speed, we conducted time-dependent studies (Figure 6), which revelaed that the host–guest interaction commences promptly upon adding sulfosulfuron to the ligand. Remarkably, within just 20 s we observed a substantial decrease in the fluorescence enhancement, indicating the completion of the host–guest interaction within a mere 150 s.

## Computational Study

### Geometry Optimization and complexation energy

Geometry optimization is a computational chemistry method that finds the lowest-energy structure of a molecule. It is a type of energy minimization, which is the process of finding the lowest-energy configuration of a system given a set of constraints. The energy that we get from geometry optimization is the total energy of the molecule at its optimized geometry. Table 3 shows the optimization energies of the ligand SS and the complex CTCHN3PPh_SS.

**Table 3:** Geometry Optimization and Complexation Energy of Sulfosulfuron and CTCHN3PPh_SS Complex

basis set	method	gas	solvent	difference
optimization details of sulfosulfuron (SS)				
b3lyp	6-31g (d,p)	–2271.89	–2271.930538	0.036724
b3lyp	6-311g (d,p)	–2272.3	–2272.33927	0.03946
cam b3lyp	6-31g (d,p)	–2271.24	–2271.278204	0.037843
cam b3lyp	6-311g (d,p)	–2271.65	–2271.692004	0.040777
optimization details of the CTCHN3PPh_SS complex				
b3lyp	6-31g (d,p)	–7313.4	–7313.485067	0.084874
b3lyp	6-311g (d,p)	–7314.95	–7315.045813	0.093284
cam b3lyp	6-31g (d,p)	–7311.88	–7311.974421	0.091938
cam b3lyp	6-311g (d,p)	–7311.88	–7311.974421	0.091955

### Molecular Docking and Dynamics

**Figure 7:**
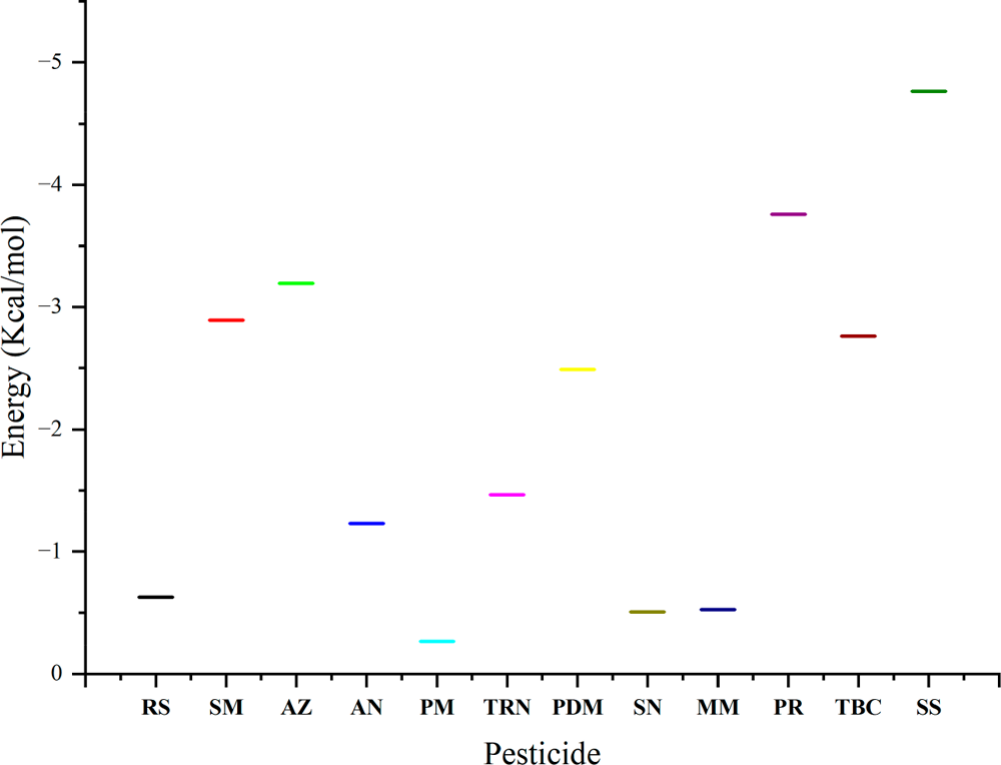
Docking scores of CTCHN3PPh with different pesticides.

**Figure 8:**
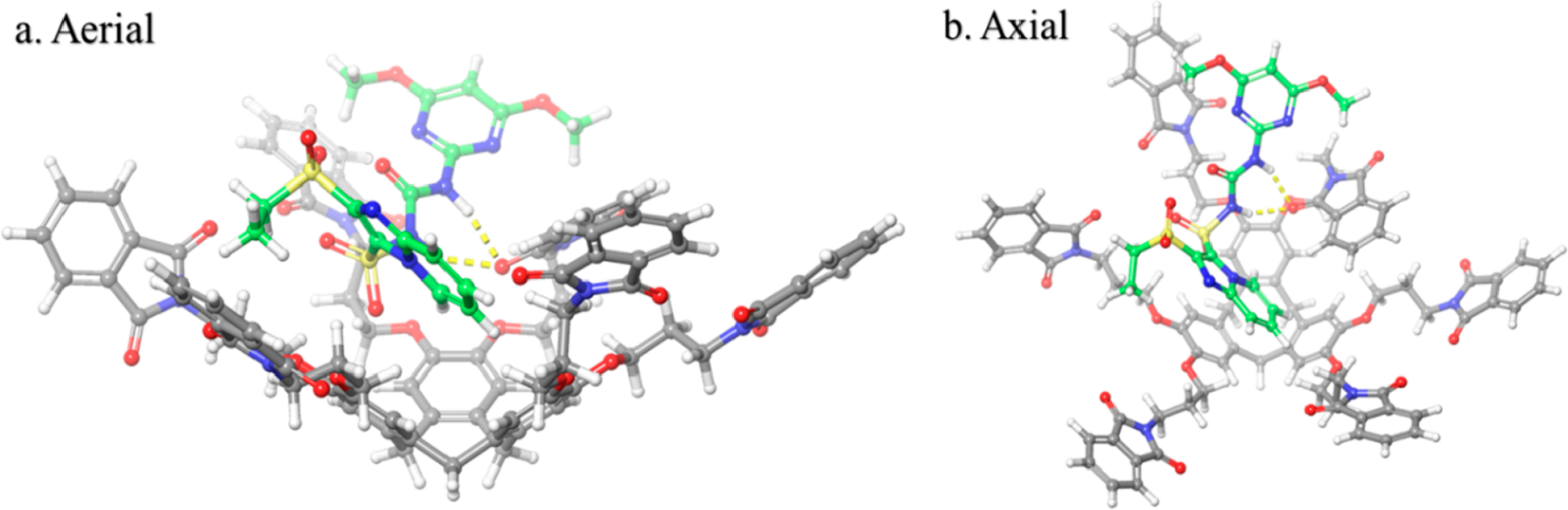
Docking interaction of host CTCHN3PPh and guest sulfosulfuron (the yellow dotted line indicates the hydrogen bonding).

**Figure 9:**
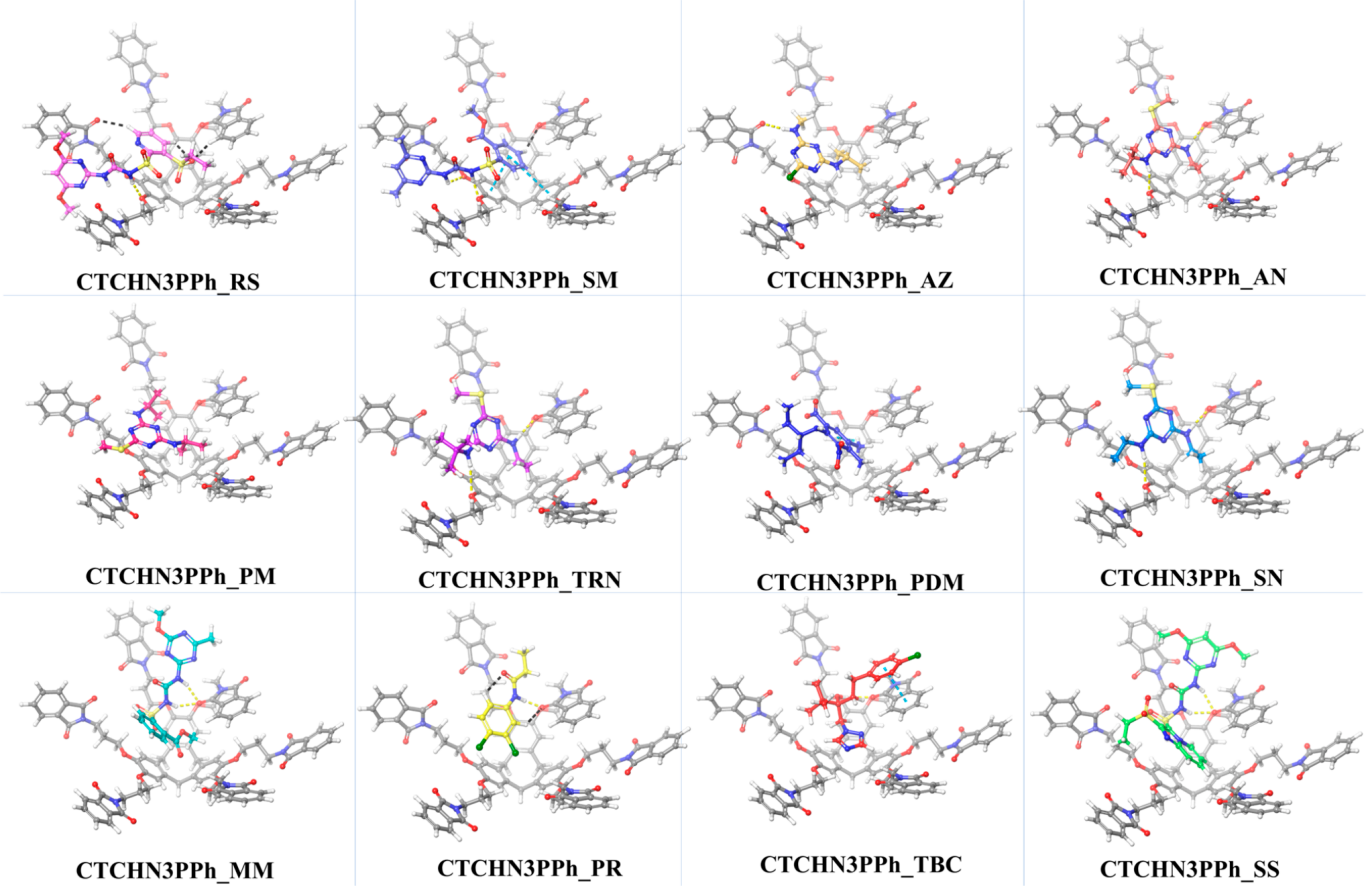
Docking poses of CTCHN3PPh with different pesticides

To obtain effective binding interactions, the receptor was optimized and allowed to participate in docking studies with all possible conformers of the guest analytes (Figure 9). The analytes were then ranked based on their docking scores, and the most favorable analyte was selected for further studies. The best docking complex was selected based on the docking score (Figure 7), which reflects the strength of interaction between the host and guest molecules. The results of the docking study showed that sulfosulfuron had the highest docking energy, indicating that it can form stable complexes with CTCHN3PPh (Figure 7). The molecular interactions responsible for this strong binding involve aromatic hydrogen bonds between CTCHN3PPh and the analyte, as well as intramolecular interactions within the analyte molecule (Figure 8).

Overall, the findings of the present study suggest that sulfosulfuron is a promising analyte for forming stable complexes with CTCHN3PPh. The insights gained from the molecular docking study can be further used to design and develop new materials with improved properties.

**Figure 10:**
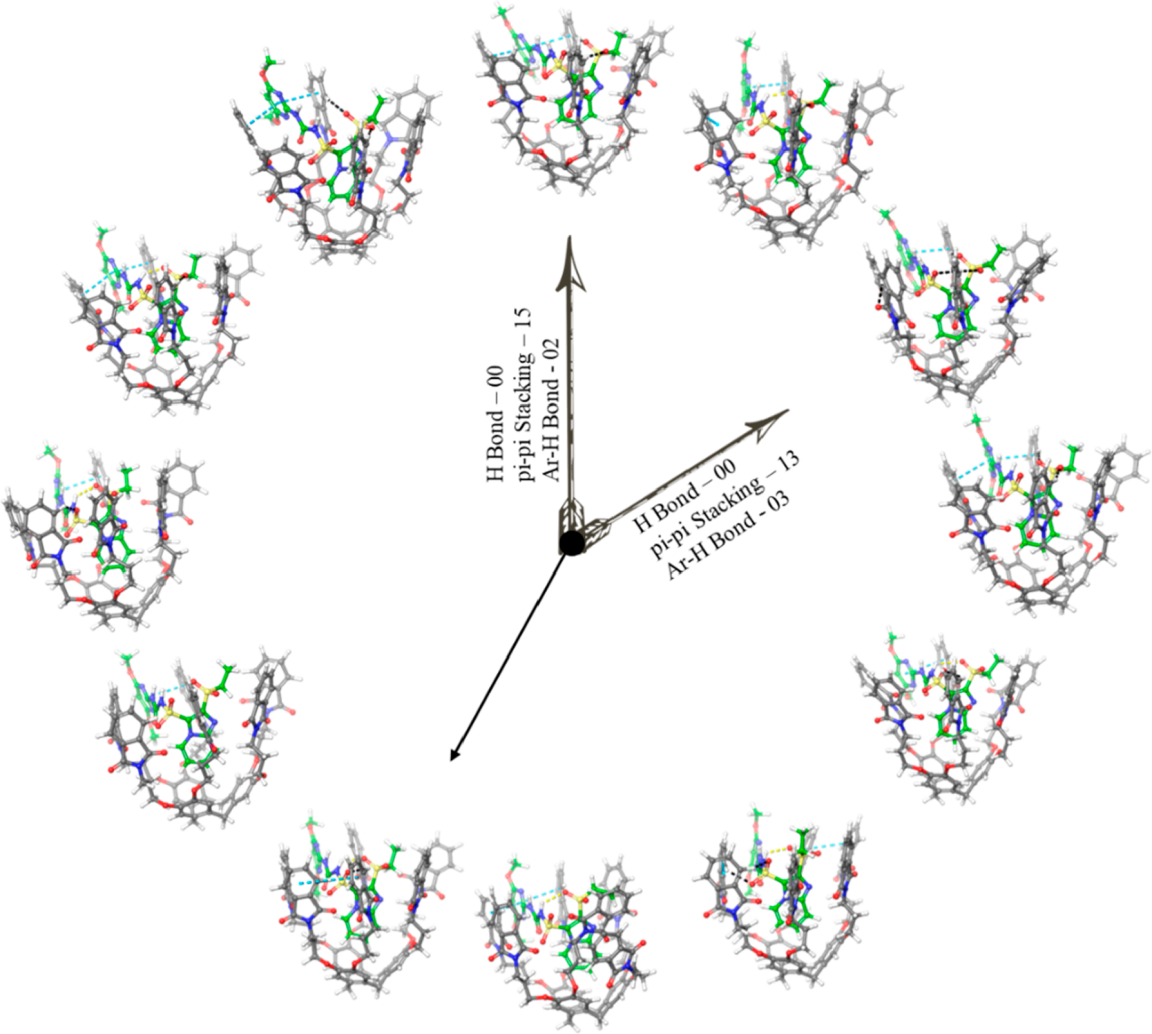
Molecular dynamics poses of CTCHN3PPh and SS at different time intervals.

Using the Schrodinger Desmond software, a MD simulation of the CTCHN3PPh and sulfosulfuron complex was conducted for 100 ns. Throughout the time, we observed some major significant interactions at different time frame, as shown in Figure 10 and Table 4. The simulation resulted in the emergence of intermolecular H-bonds and van der Waals forces. The most significant interaction is the one at the pose observed at 28 ns; in this interaction, we observed the π–π stacking and aromatic hydrogen bond between the carbonyl group of sulfosulfuron and the aromatic phthalimide functionalization of the ligand as a host–guest interaction. The best simulation conformation was further studied using density functional theory (DFT) to gain more insight into the impact of functional group interactions. A simulation video is provided in the Supporting Information.

**Table 4:** Different Intermolecular Interactions Observed in the Molecular Dynamics Study at Different Time Intervals

frame	time	H bond	π–π Stacking	Ar–H bond
1	12 ns	1	2	1
**2**	**28 ns**	**0**	**1**	**3**
3	47 ns	0	2	2
4	52 ns	1	1	1
5	58 ns	1	2	2
6	70 ns	1	2	0
7	86 ns	1	2	1
8	89 ns	0	1	0
9	94 ns	1	1	1
10	97 ns	1	2	1
11	99 ns	0	2	2
12	101 ns	0	2	2

### NBO Analysis

In the context of a host–guest interaction between a ligand and an analyte for a fluorescence sensor, NBO analysis can provide insight into the nature of the bonding interactions between the two molecules. Specifically, NBO analysis can be used to calculate the charge transfer between the ligand and the analyte, as well as the extent to which the electron density is delocalized between the two molecules.^42,43^

**Figure 11:**
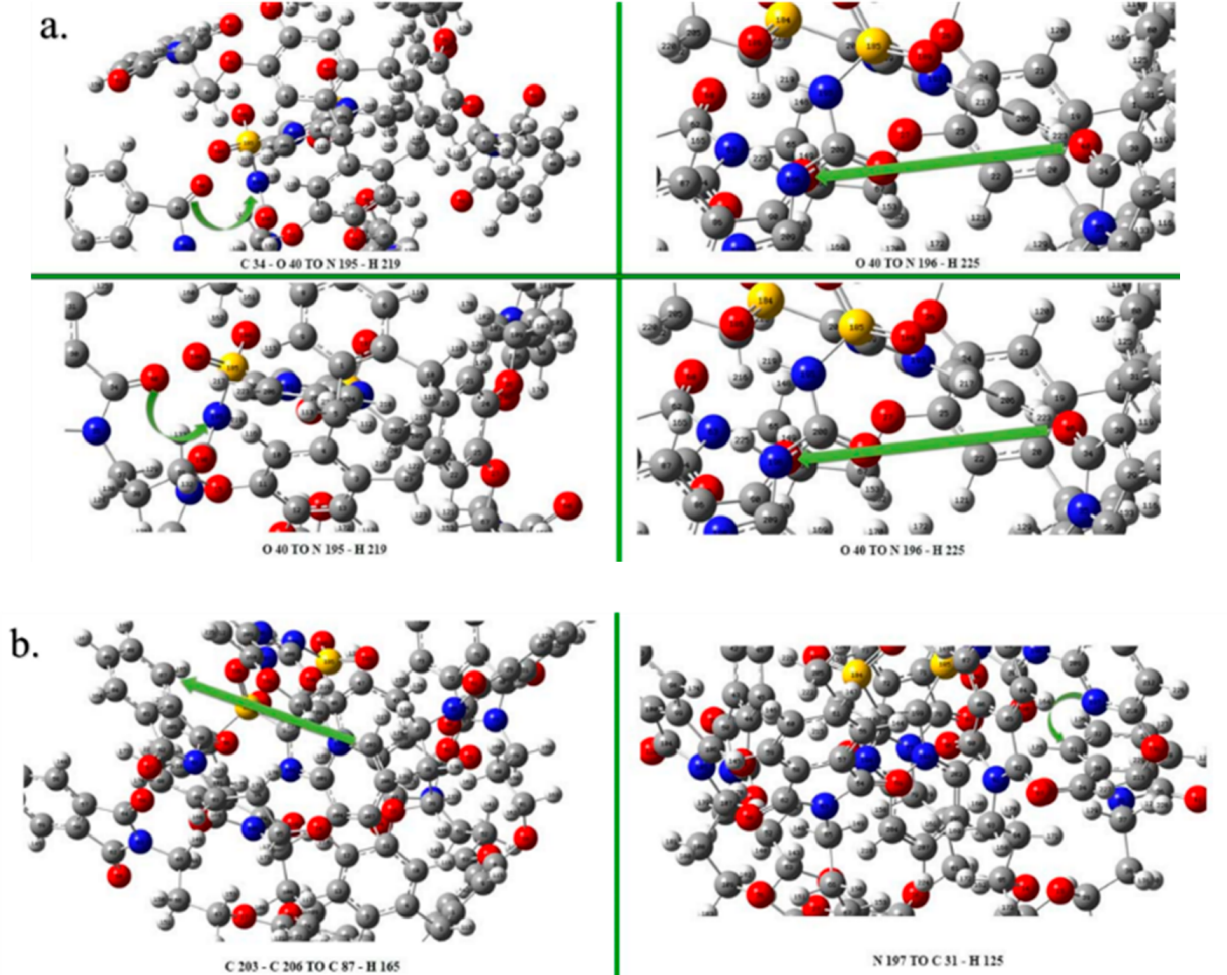
NBO analysis of CTCHN3PPh and sulfosulfuron (SS). (a) Donor atom from CTCHN3PPh, acceptor atom from SS, (b) Donor atom from SS, acceptor atom frm CTCHN3PPh).

The NBO analysis of the donor–acceptor interactions between the CTCHN3PPh ligand and sulfosulfuron analyte reveals significant interactions. First, there is a σ–σ* interaction between the C–O bond in CTCHN3PPh and the N–H bond in sulfosulfuron, indicating substantial overlap between their σ bond orbitals (Figure 11a). Additionally, the lone pair on the oxygen atom in CTCHN3PPh donates electron density to the σ* antibonding orbital of the N–H bond in sulfosulfuron, forming an LP to σ* interaction. Furthermore, there are LP to σ* interactions involving O40 in CTCHN3PPh and different N–H bonds in sulfosulfuron (Figure 11a). In the opposite interaction, sulfosulfuron acts as the donor and CTCHN3PPh as the acceptor, resulting in a σ to σ* interaction between the C–C bond in sulfosulfuron and the C–H bond in CTCHN3PPh, along with an LP to σ* interaction between the lone pair on the N atom in sulfosulfuron and the σ* antibonding orbital of the C–H bond in CTCHN3PPh, as shown in Figure 11b. These interactions provide valuable insights into the strength and nature of the donor–acceptor interactions within the fluorescence sensor system. NBO analysis of CTCHN3PPh and sulfosulfuron is demonstrated in Table 5.

**Table 5:** NBO Analysis of CTCHN3PPh and Sulfosulfuron

donor atom	acceptor atom		
CTCHN3PPh	sulfosulfuron	energy (kcal/mol)	interaction^a^
C34–O40	N195–H219	3.05	σ to σ
O40	N196–H225	5.17	LP to σ
O 40	N195–H219	1.29	LP to σ
O40	N196–H225	4.06	LP to σ

donor atom	acceptor atom		
sulfosulfuron	CTCHN3PPh	energy (kcal/mol)	interaction^a^
C203–C206	C87–H165	0.3	σ to σ
N197	C31–H125	0.37	LP to σ

a LP indicates lone pair.

### DFT Study and Other Molecular Properties

Host–guest complexation studies use molecular properties such as hardness, softness, chemical potential, and electrophilicity index to understand the stability, reactivity, and electronic properties of the ligand, analyte, and complex. These properties can help predict the charge transfer mechanisms and reactivity of the system and design better host–guest systems.^44−46^ A molecule with a lower chemical potential is more likely to act as a nucleophile. Hardness values show how resistant a molecule is to charge transfer, while softness values measure a molecule’s susceptibility. The complex’s hardness value is intermediate between the values of the ligand and analyte alone, indicating some intermediate stability and reactivity. We can observe that the ligand has a higher chemical potential value (−3.9645) than the analyte (−4.2725), suggesting that the ligand is more likely to act as a nucleophile (Table 6, Figure S7). The energy gap (*E*_g_) value for the complex formed between the ligand and the analyte is smaller than those of the individual components. A smaller energy gap indicates that the complex may have increased stability and reactivity compared to the individual components. This is because a smaller energy gap implies that electrons can be more easily excited from the HOMO to the LUMO, leading to a higher likelihood of charge transfer. The calculated values of molecular properties are shown in the table below.

**Table 6:** Other Molecular Properties Calculated from the DFT Study

	HOMO	LUMO	*E*_g_ (eV)	hardness	softness	chem. potential	electrophilicity index
CTCHN3PPh	–5.60	–2.32	3.28	1.64	0.30	–3.96	4.78
SS	–6.70	–1.83	4.87	2.43	0.20	–4.26	3.73
CTCHN3PPh_SS	–5.40	–2.60	2.80	1.40	0.35	–4.00	5.71

**Figure 12:**
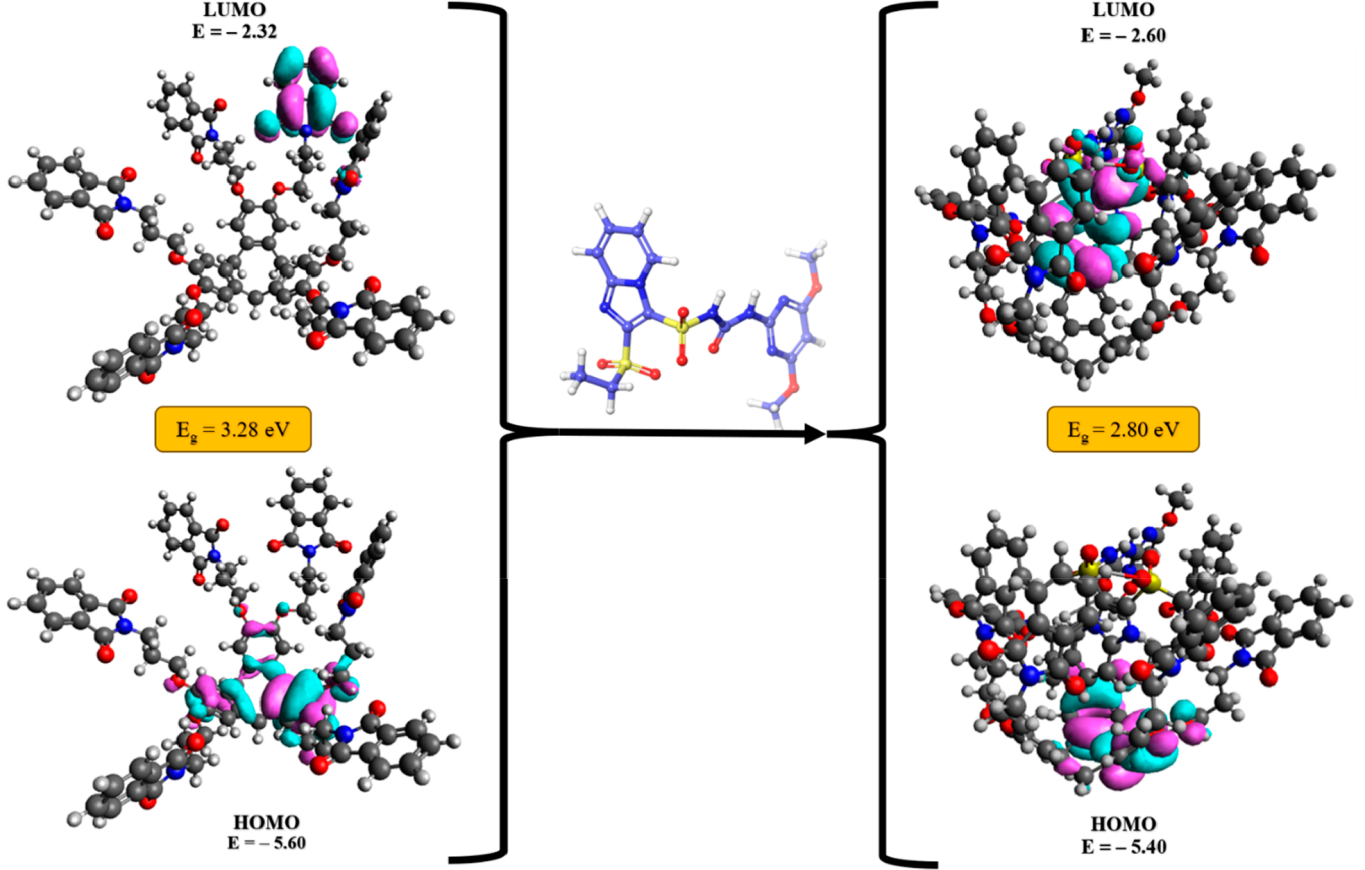
HOMO to LUMO transition in the CTCHN3PPh and CTCHN3PPh_SS complexes.

In the DFT study, it was observed that charge transfer was occurring between the CTCHN3PPh and sulfosulfuron in the host–guest complex. Specifically, electrons were being transferred from the highest occupied molecular orbital (HOMO) of CTCHN3PPh to the lowest unoccupied molecular orbital (LUMO) of sulfosulfuron (Figure 12). This charge transfer phenomenon is an important aspect of the binding process between the host and guest molecules, as it can lead to changes in the electronic properties and results in fluorescence enhancement. This observation suggests that the charge transfer mechanism may contribute to the stability and reactivity of the host–guest complex, and it highlights the importance of understanding the electronic properties of the complex in predicting and interpreting its behavior in chemical reactions.

### Binding Behavior

**Figure 13:**
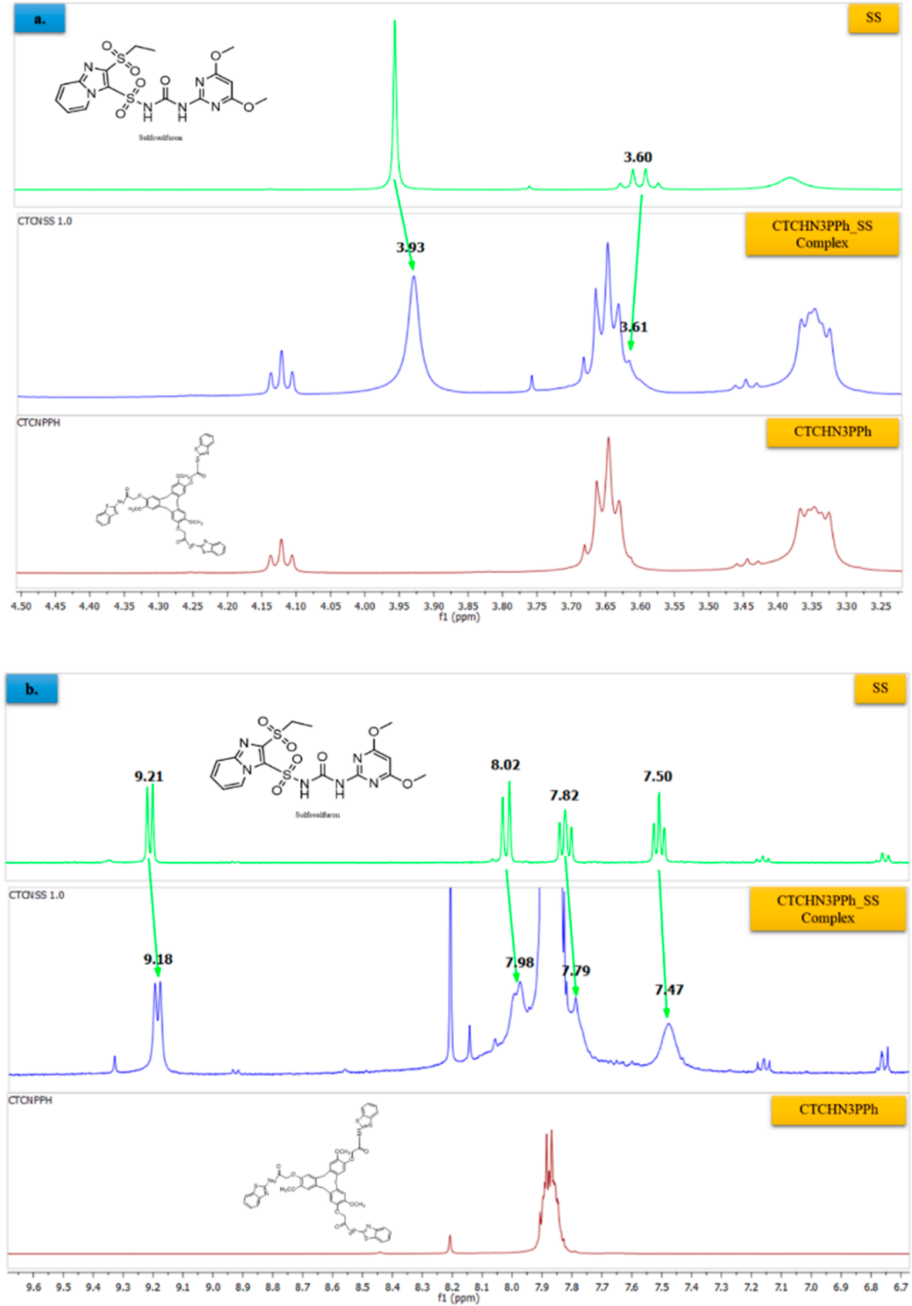
NMR Analysis of the CTCHN3PPh_SS complex. (a) Changes in the aliphatic CH_2_ peaks of CTCN3PPh with SS. (b) Changes in the aromatic region peak of CTCN3PPh with SS.

The detailed investigation into binding behavior obtained through computational study is corroborated through NMR complexation study. The major observations made into NMR complexation is detailed in Figure 13a and b. The evidential shift of aromatic region in sulfosulfuron is shown in Figure 13b. The aromatic region peak is shifted to 9.18 from 9.21 ppm, 8.02 from 7.98 ppm, and 7.82 from 7.79 ppm; these shifts are aligned with our molecular docking results, where we observed π–π stacking between the aromatic ring of sulfosulfuron and the ligand. Another interaction observed through the NMR complex study is the shifting of the aliphatic proton in sulfosulfuron. This interaction shows that the analyte is coordinating through van der Waals forces and/or hydrophobic interactions. This binding behavior observed in NMR complexation study is in very good agreement with the computational findings.

The changes in the peaks of the aromatic region of both the ligand and the analyte as well as the changes in the aliphatic CH_2_ peaks indicate that there are likely to be intermolecular interactions occurring between the ligand and the analyte in the complex. The changes in the aromatic peaks could indicate that there are π–π stacking interactions occurring between the aromatic rings of the ligand and analyte. The changes in the aliphatic CH_2_ peaks (3.60 to 3.61 ppm) (Figure 13a) could indicate that there are van der Waals forces and/or hydrophobic interactions occurring between the aliphatic groups of the ligand and analyte.

### Binding Phenomena

The investigation into the binding phenomena between the synthetic CTCHN3PPh receptor and the pesticide sulfosulfuron is a testament to the power of multidisciplinary research. The combined results from various analytical techniques, including NBO analysis, DFT calculations, molecular docking, and NMR experiments, mutually corroborate and complement each other, offering a comprehensive and cohesive view of the intricate host–guest interaction.

#### Charge Transfer and π–π Stacking

The DFT and NBO studies unveil a profound charge transfer phenomenon between the receptor and sulfosulfuron, indicating electron flow from the HOMO of CTCHN3PPh to the LUMO of sulfosulfuron. This charge transfer mechanism, supported by computational evidence, lays the foundation for understanding the electronic aspects of the binding. Furthermore, this charge transfer is intimately connected to the observed π–π stacking interactions, as electrons moving between molecular orbitals underpin the aromatic interactions discerned through NMR and docking studies.

#### Molecular Docking and Molecular Dynamics

The molecular docking results, which position sulfosulfuron as the most favorable analyte for complex formation, resonate with the charge transfer mechanism identified in the DFT and NBO analyses. These docking findings are underpinned by NMR titration experiments, where changes in the aromatic regions of both the ligand and the analyte are observed, strongly suggesting π–π stacking interactions. Furthermore, molecular dynamics simulations corroborate the complex’s stability, emphasizing the persistence of the intermolecular interactions and providing a dynamic perspective that complements the static docking results.

#### Experimental Validation Through NMR

NMR titration experiments serve as an invaluable experimental validation of the binding interactions. The changes observed in the aromatic regions of both the ligand and analyte, along with shifts in the aliphatic CH_2_ peaks, directly support the presence of π–π stacking interactions, hydrophobic interactions, and van der Waals forces, aligning with the predictions from computational studies and docking.

In essence, these diverse methodologies seamlessly converge to create a unified narrative of binding phenomena. The charge transfer mechanisms, aromatic interactions, intermolecular forces, and stability of the complex are consistently reaffirmed through various analytical lenses. This holistic approach not only deepens our understanding of the host–guest interaction but also exemplifies the synergy achieved through interdisciplinary research. It is through this collective effort that we gain profound insight into the intricate binding behavior between the CTCHN3PPh receptor and sulfosulfuron, paving the way for further advancements in supramolecular chemistry and molecular recognition studies.

### Water Sample Analysis

Analyte detection in environmental samples was accomplished in the 50% DMF/water solvent system. For the tests, a standard spiking method was used. Water samples were spiked with different concentrations, and the results obtained were in good agreement with experimental data. Real sample analysis is carried out from http://www.realsamplesolution.co.in, The results obtained show the recovered concentrations are in agreement with desired concentration. Results of water samples analysis are given in Table 7.^47^

**Table 7:**
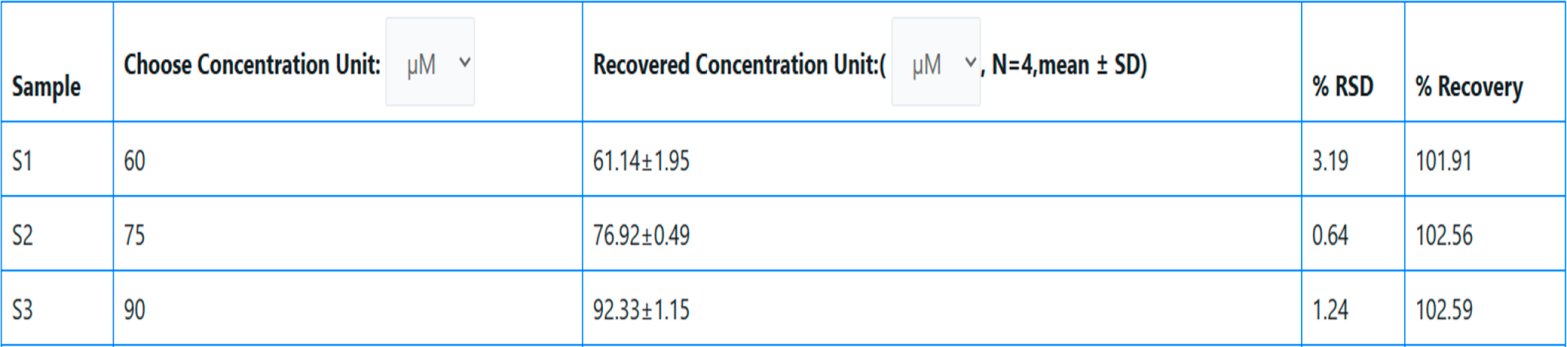
Water Sample Analysis of CTCHN3PPh_SS

## Conclusion

Our study has demonstrated the remarkable potential of phosthalimide-functionalized cyclotricatechylene (CTCHN3PPh) as a sulfosulfuron fluorescence sensor, providing a practical means of identifying the sulfosulfuron pesticide. We validated the charge transfer, hydrogen bonding, and π–π stacking interactions involved in the binding mechanism using a comprehensive approach combining computational and experimental studies. We also established accurate and reliable measurements for the binding constant (1.7 × 10^5^) and the limit of detection (58 μM) with a 1:1 stoichiometry. Additionally, despite difficult circumstances with the 50% water content, our investigation has successfully demonstrated the practical applicability of the CTCHN3PPh sensor in accurately detecting sulfosulfuron in real sample analysis. This important result demonstrates the sensor’s potential as a useful fluorescence probe for accurate pesticide detection applications. Overall, our research demonstrates the significant benefits of using molecular sensing methods with receptors based on cyclotriveratrylene in the study of pesticides. The discovery of effective and sensitive detection techniques for various agricultural compounds is made possible by this work.

## Experiments

### Chemicals and Instruments

All the solvents used in the synthesis were commercially available and were used as received. All compounds, including 4-aminoacetophenone, pyrrole, methane sulfonic acid, K_2_CO_3_, KI, and 2-chloroacetamide, were purchased from Sigma-Aldrich. Merck supplied fluorescence-active TLC plates (F-254). A magnetic stirrer (REMI-5MLH) and a micropipette (VAR VOL 100–1000 μL, Kasablanka-Mumbai) were utilized. Before use, all glassware was meticulously calibrated. Uncorrected melting points were determined using a VEGO model VMP-DS (Mumbai, India). Using a micromass Quarter2 instrument, electrospray ionization (ESI) mass spectra (MS) were collected (IISER, Pune). NMR spectra were recorded on a two-channel 400 MHz NMR spectrometer (Brüker Biospin, Switzerland; Indrashil University, Mehsana). A SCHIMADZU-1900 system was used to record UV–vis spectra (Ganpat University). Fluorescence spectra were acquired on a Jasco FP-6500 spectrofluorimeter (Gujarat University).

### General Procedures for the UV–vis and Fluoroscence Measurements

**Figure 14:**
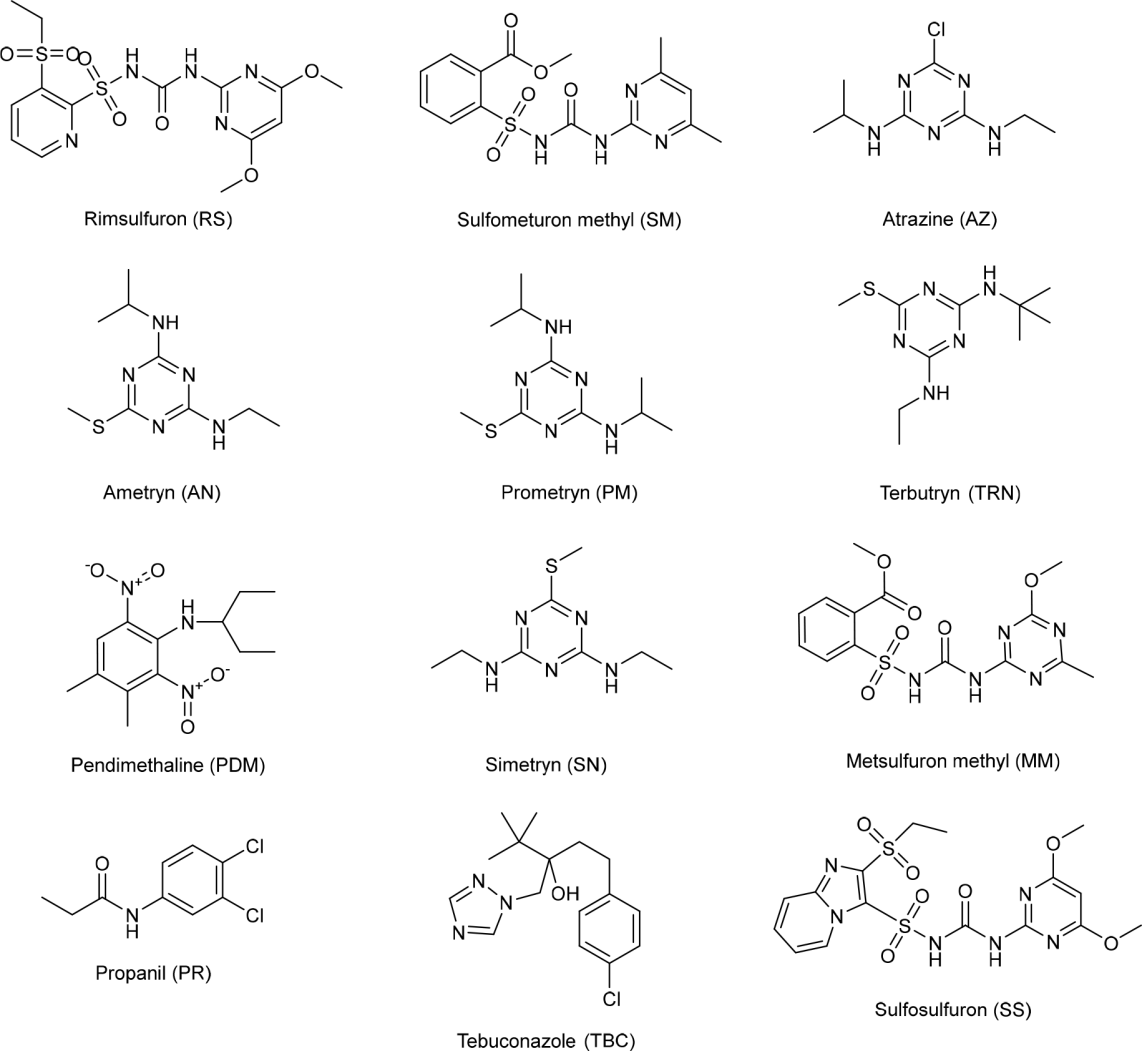
Structures of Various Pesticides.

The ability of CTCHN3PPh to sense pesticides was investigated using spectrophotometric and spectrofluorometric measurements. To prepare for the spectroscopic studies, a stock solution of the receptor CTCHN3PPh (2 mM) was made using DMF as the solvent and then diluted to 20 mM. Similarly, stock solutions of various pesticides (2 mM), including rimsulfuron (RS), sulfometuron methyl (SM), atrazine (AZ), ametryn (AN), prometryn (PM), terbutryn (TRN), pendimethaline (PDM), simetryn (SN), metsulfuron methyl (MM), propanil (PR), tebuconazole (TBC), and sulfosulfuron (SS) (Figure 14), were prepared in DMF. The change in the absorption band of CTCHN3PPh (10 μM) upon the addition of various pesticides was recorded in the UV-A and visible range (200–800 nm). The receptor was excited at 379 nm, and the change in emission maxima was observed upon the addition of the mentioned analytes. The temperature was maintained at 298 ± 2 K throughout the study, and the excitation and emission slit widths were set at 5 nm for all measurements.

#### Computational Study

Molecular docking, molecular simulation, and DFT studies are computational techniques used to understand the interaction between CTCHN3PPh and sulfosulfuron. Molecular docking predicts the binding of two molecules based on their 3D structures, providing information on binding orientation and interaction energy to determine the stability of the complex. Molecular simulation, on the other hand, uses mathematical models to study the behavior of molecular systems over time, providing deeper insights into the binding mechanism between CTCHN3PPh and sulfosulfuron. DFT, a quantum mechanical computational method, calculates the electronic properties and bonding information of molecules, offering further insights into the binding mechanism between the two molecules. The DFT study was carried out with Becke’s three-parameter hybrid exchange (B3LYP) method and the hybrid functional using the Coulomb attenuating method (CAM-B3LYP) with 6-31G(d,p) and 6-311G(d,p) basis sets, out of which the best optimized result was selected for further studies. These computational techniques together provide a comprehensive understanding of the binding behavior between CTCHN3PPh and sulfosulfuron and help to investigate experimental results.^48−50^

The docked complex of CTCHN3PPh and SS was subjected to a molecular dynamics simulation study using the Desmond program and the OPLS force field. To set up the system, the “System Setup” software was used, which involved solvation by TIP3P water model and neutralization of the system’s charge by counterions. The simulation was performed for 100 ns in the NPT ensemble, and various evaluative measures, such as RMSD, RMSF, and SSC, were recorded using the “Simulation Interaction Diagram” module. The trajectory file was further analyzed using the “Desmond Trajectory Clustering” tool to identify the average and most representative structure of CTCHN3PPh.

## Supporting Information Available

Synthesis of cyclotricatechylene; mass spectrum, NMRspectrum, and FTIR spectrum of CTC; mass spectrum, NMR spectrum, and FTIR spectrum of newly synthesized CTCHN3PPh; and HOMO–LUMO energy diagram of CTCHN3PPh, SS, and CTCHN3PPh_SS (PDF)
Simulation video of the conformation analysis (MPG)

## Supplementary Material

ao3c05510_si_002.pdf

ao3c05510_si_003.mpg
