# Supplementary material for: Propyl-phthalimide Cyclotricatechylene-Based Chemosensor for Sulfosulfuron Detection: Hybrid Computational and Experimental Approach
Source: ACS Omega. 2023 Oct 24;8(44):41523–36. doi: 10.1021/acsomega.3c05510 (PMC10633956; doi:10.1021/acsomega.3c05510)
Supplement: Supplementary file 1 — ao3c05510_si_002.pdf [file ao3c05510_si_002.pdf]

Propyl-Phthalimide cyclotricatechylene based chemosensor for the sulfosulfuron detection: Hybrid computational and experimental approach

Nihal Patel [a‡], Krunal Modi [b], Keyur Bhatt [a*], Jaymin Parikh [a‡], Ajay Desai [a*], Bhavesh Jain [c], Nirali Parmar [a], Chirag N. Patel [d,e], Alan Liska [f], Jiri Ludvik [f], Shibu Pillai [g], Brij Mohan [h]*

[a] Department of Chemistry, Faculty of Science, Ganpat University, Kherva-384012, Mehsana, Gujarat, India.

[b] Department of Humanity and Sciences, Indrashil university, Kadi, Mehsana-382740, Gujarat, India.

[c] Department of Computer Science & Engineering, Indrashil University, Kadi, Mehsana-382740, Gujarat, India.

[d] Department of Botany, Bioinformatics and Climate Change Impacts Management, School of Science, Gujarat University, Ahmedabad 380009, India.

[e] Biotechnology Research Center, Technology Innovation Institute, Abu Dhabi 9639, United Arab Emirates.

[f] Department of Molecular Electrochemistry and Catalysis, J. Heyrovsky Institute of Physical Chemistry, Academy of Sciences of the Czech Republic, Dolejskova 2155/ 3,182 23 Praha 8, Czech Republic.

[g] Department of Chemistry, Institute of Technology, Nirma University, Ahmedabad 380009, Gujarat, India

[h] Centro de Química Estrutural, Institute of Molecular Sciences, Instituto Superior Técnico, Universidade de Lisboa, Av. Rovisco Pais, 1049-001 Lisboa, Portugal.

Figure S1 FTIR of Cyclotricatechylene (CTC)

Figure S2 1H NMR of Cyclotricatechylene (CTC)

Figure S3 Mass of Cyclotricatechylene (CTC)

Figure S4 1H NMR of CTCHN3PPh

Figure S5 Mass of CTCHN3PPh

Figure S6 FTIR of CTCHN3PPh

Figure S7 HOMO LUMO energy diagram

Scheme S1 Synthesis of Cyclotricatechylene (CTC)

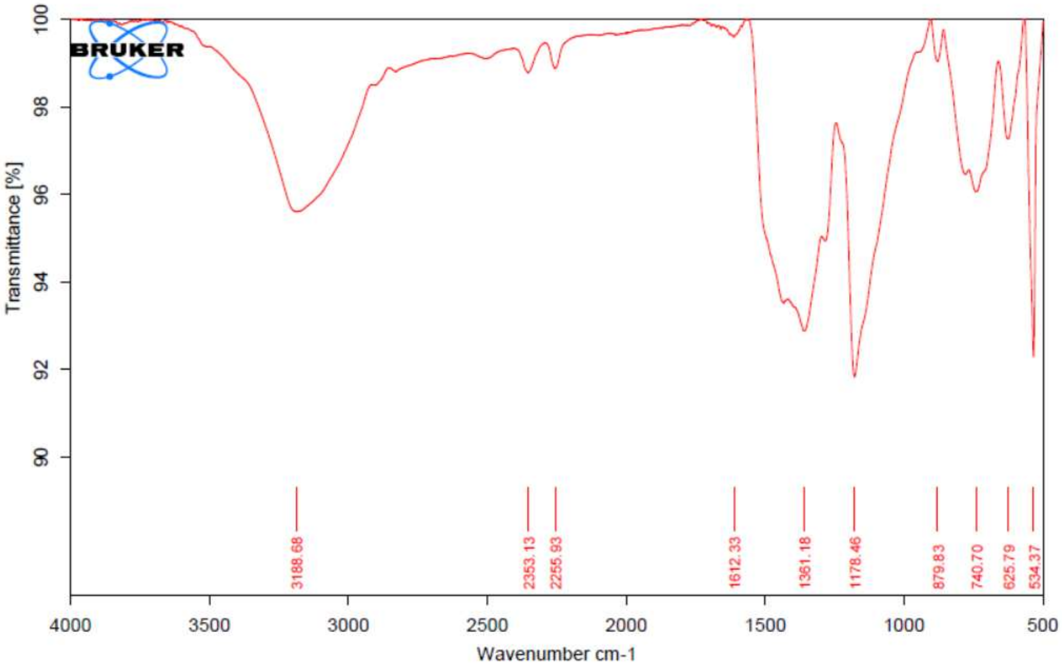

Figure S1. FTIR of Cyclotricatechylene (CTC)

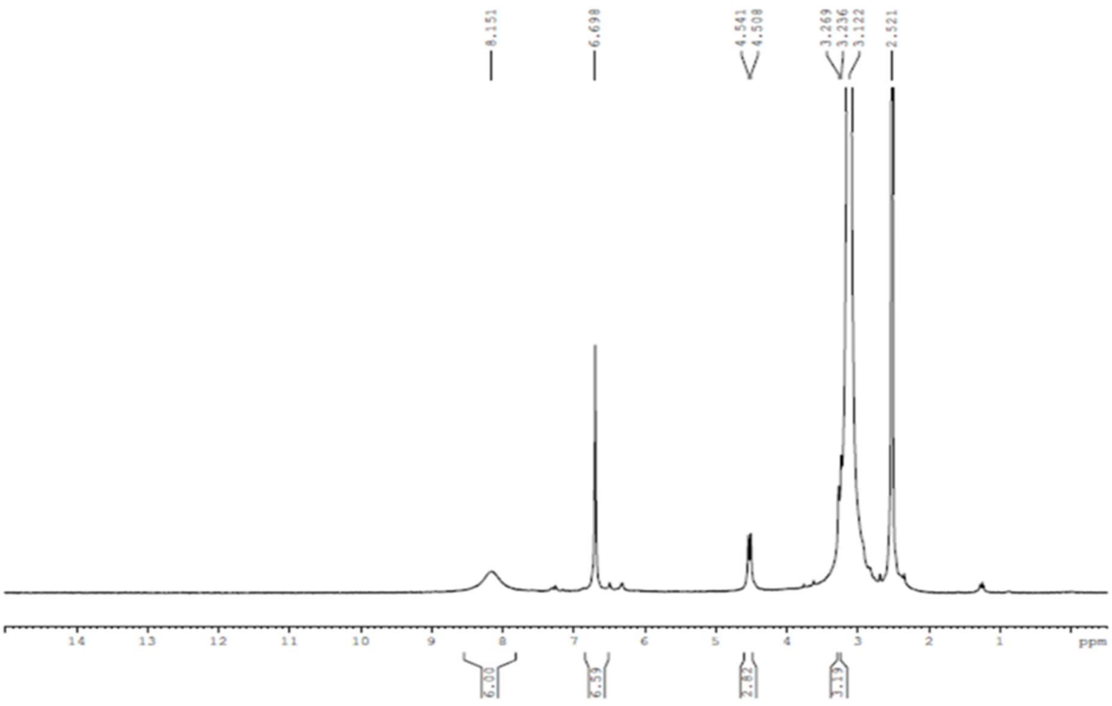

Figure S2. 1H NMR of Cyclotricatechylene (CTC)

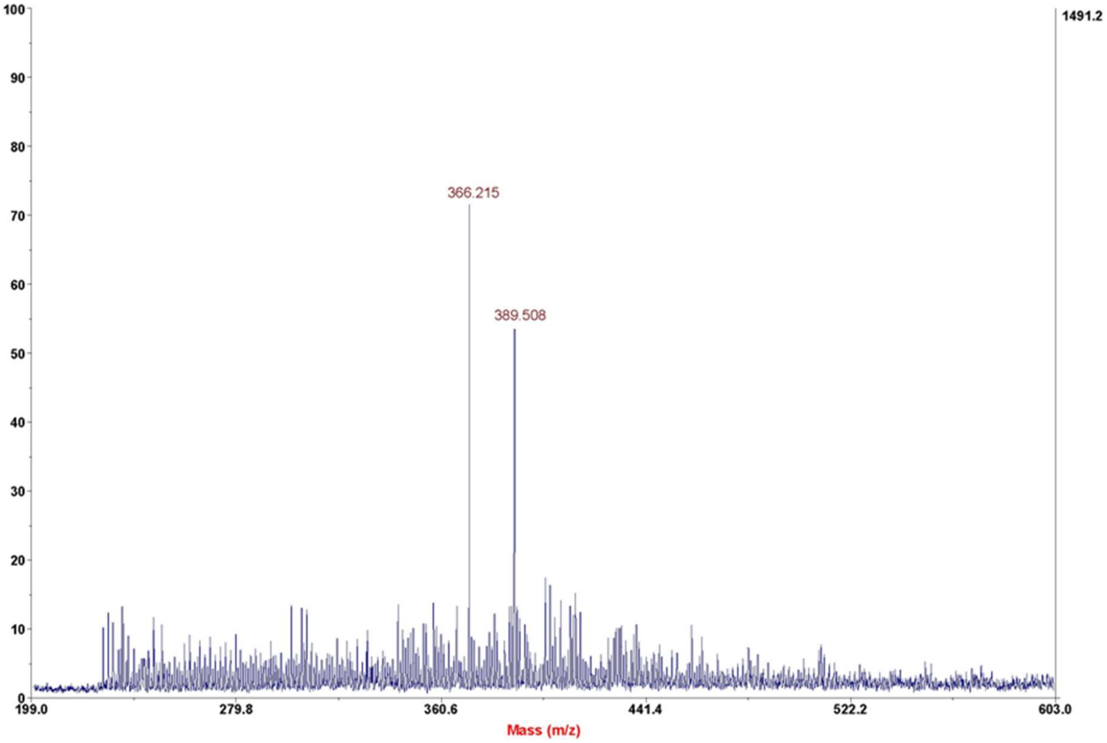

Figure S3. Mass of Cyclotricatechylene (CTC)

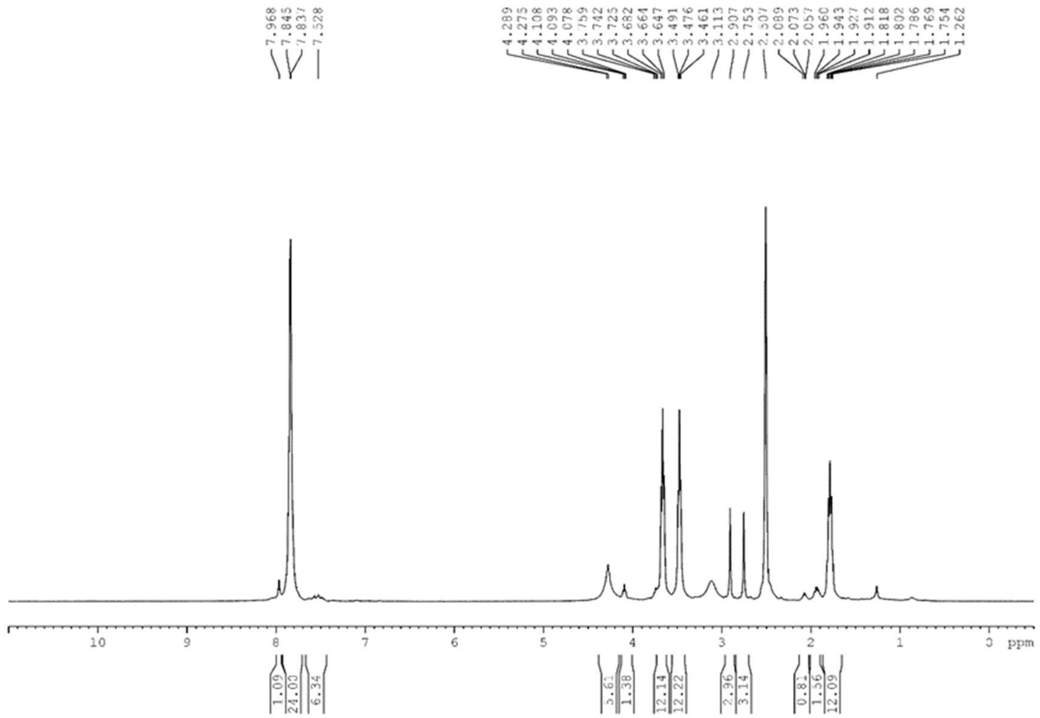

Figure S4. ^{1}H NMR of CTCHN3PPh

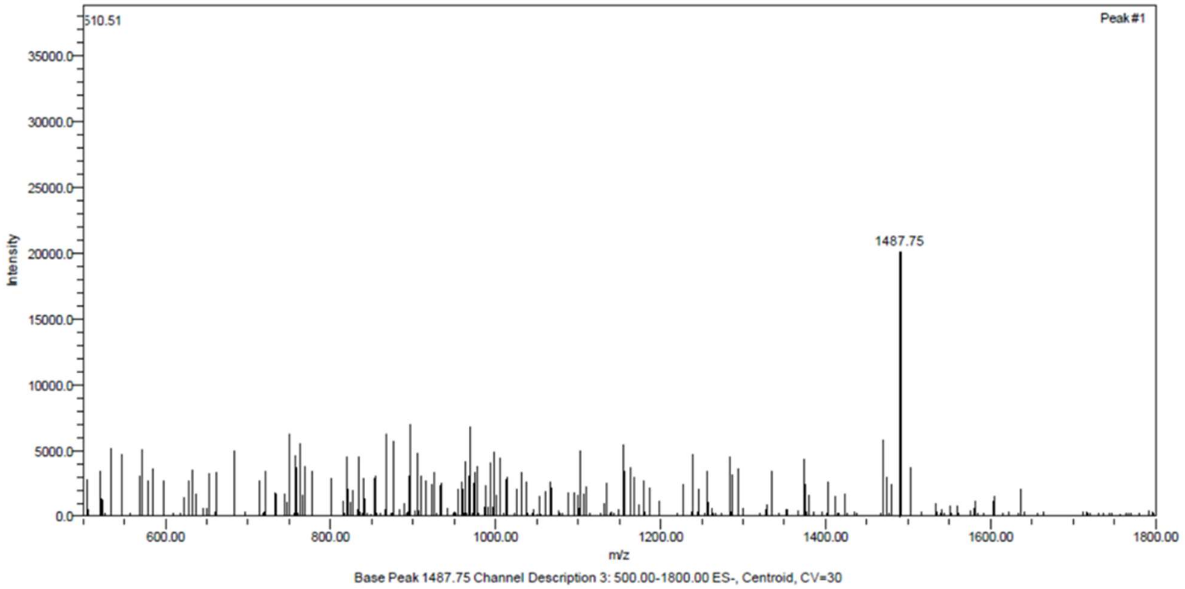

Figure S5. Mass of CTCHN3PPh

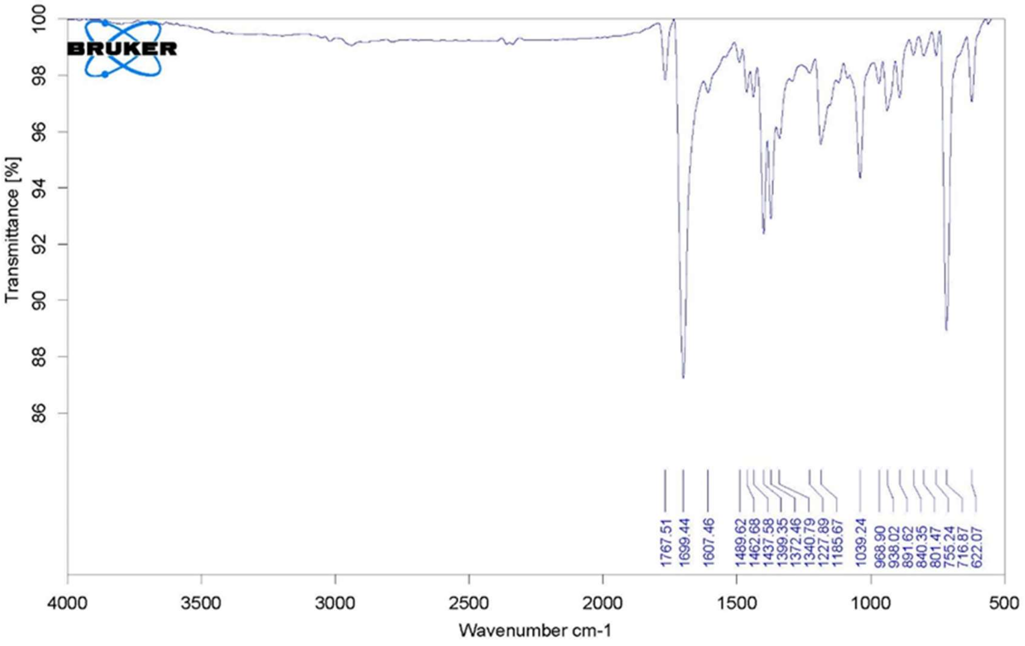

Figure S6. FTIR of CTCHN3PPh

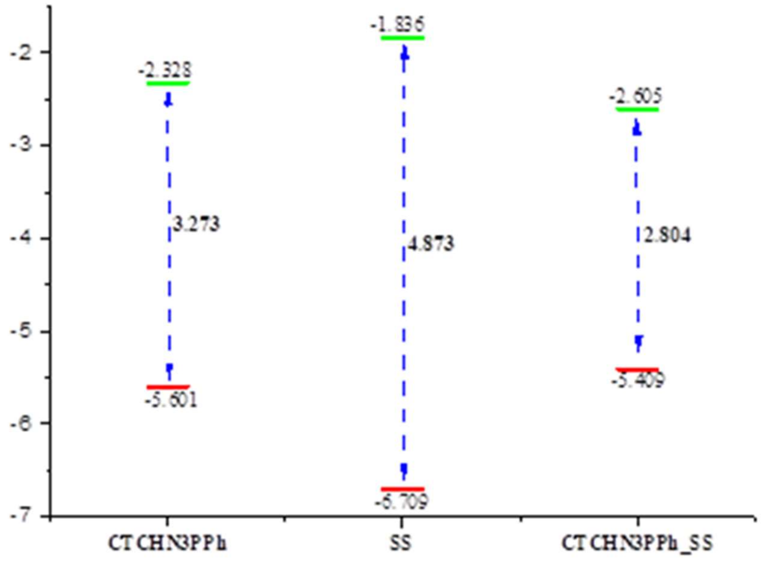

Figure S7. HOMO LUMO energy diagram

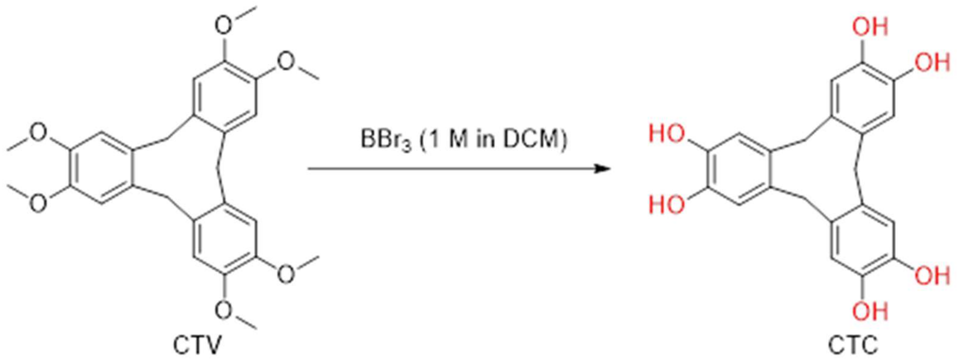

Scheme S1. Synthesis of Cyclotricatechylene (CTC)
